# Motivations of Childbearing Among Iranian Working Mothers With Two or More Children: A Qualitative Study

**DOI:** 10.1002/hsr2.72831

**Published:** 2026-07-16

**Authors:** Neda Asadi, Jamileh Farokhzadian, Nouzar Nakhaee, Sirous Pourkhajoei

**Affiliations:** ^1^ Student Research Committee Kerman University of Medical Sciences Kerman Iran; ^2^ Nursing Research Center Kerman University of Medical Sciences Kerman Iran; ^3^ Social Determinants of Health Research Center, Institute for Futures Studies in Health Kerman University of Medical Sciences Kerman Iran

**Keywords:** childbearing intentions, fertility motivation, Iran, working mothers

## Abstract

**Background and Aim:**

Iran, like many other countries, faces declining population growth and concerns about aging. Rising female participation in higher education and the labor market has positioned education and employment as factors that may conflict with childbearing. Most studies on fertility motivations have focused on childless individuals or one‐child families, while little attention has been given to larger families. This study aimed to explore the motivations of childbearing among Iranian working mothers with two or more children.

**Method:**

This qualitative content analysis was conducted among 27 working mothers affiliated with Kerman University of Medical Sciences, in southeastern Iran. Participants were selected through purposive sampling with maximum variation. Inclusion criteria required participants to be married for ≥ 5 years, have ≥ 2 biological children, and express intent for further childbearing. Data were collected via semi‐structured interviews and Guba and Lincoln's criteria were applied to increase the trustworthiness and rigor of the study. Data were analyzed using Graneheim and Lundman's method and MAXQDA 2020.

**Results:**

The motivations of working women with two children in childbearing are comprehensive, and four main themes emerged from the responses: (1) positive reproductive memories and support, (2) concerns about single‐child outcomes, (3) personal and religious beliefs, and (4) demographic concerns and pronatalist influences.

**Conclusion:**

Fertility intentions among working women in Iran are shaped by emotionally embedded and culturally situated factors. Understanding these motivations is essential for designing responsive and targeted population policies. The findings support the need for mixed‐method research to deepen theoretical insight and enhance generalizability across regions and contexts.

## 1 Introduction

Population dynamics and fertility behavior are shaped by a complex interplay of biological, economic, social, cultural, and institutional factors. Fertility intentions are widely recognized as a central component of population science and a key indicator for demographic forecasting and social policy development [1, 2]. Marriage, family formation, and the desire to have children are among the most influential drivers of population change. Fertility decisions are shaped not only by individual preferences but also by broader social norms, economic conditions, and policy environments [3].

Historically, demographic research emphasized biological and economic determinants of fertility behavior, often overlooking cultural and gendered dimensions. Recent scholarship has increasingly highlighted the role of social values, gender norms, and institutional contexts in shaping reproductive behavior [4, 5]. In recent decades, demographic research has moved beyond purely economic or biological explanations to incorporate cultural values, gender norms, and institutional contexts that shape reproductive decisions. This shift aligns with the Second Demographic Transition (SDT) theory, which emphasizes fertility postponement, individual autonomy, and changing family structures [6, 7]. Simultaneously, work–family conflict models highlight how women's employment interacts with institutional supports such as paid maternity leave, flexible schedules, and childcare access to influence the feasibility and timing of childbearing [8].

Fertility decisions are major life events that influence health, economic stability, and family well‐being. Studies have identified diverse motivations for childbearing, including emotional fulfillment, cultural expectations, and perceived continuity of family lineage [9, 10]. However, recent demographic trends indicate a decline in fertility desires, with many couples opting for only one child. This shift has contributed to population aging and a disruption in the age structure, raising concerns about long‐term socioeconomic sustainability [11, 12]. Despite this decline, some couples still consider larger families ideal [13]. Frameworks such as the Second Demographic Transition theory and work–family conflict models are essential for understanding fertility intentions among working women, particularly in contexts where labor policies are inconsistently enforced.

Iran has experienced a sharp decline in fertility rates, falling below replacement level, which has raised concerns about population aging and long‐term demographic sustainability [14]. While several domestic studies have explored fertility preferences, most focus on childless couples or families with one child [15, 16, 17]. There remains a lack of in‐depth understanding of the motivations of working mothers who already have two children and are considering further childbearing. Existing studies often overlook this pivotal group, despite their importance in reversing fertility decline.

Working women face unique constraints. Employment‐related factors such as job insecurity, occupational stress, and lack of institutional support can significantly influence their reproductive decisions. In Iran, working women tend to desire fewer children than their non‐working counterparts, largely due to time constraints, economic pressures, and limited access to supportive workplace policies [18, 19, 20]. Several qualitative studies among Iranian working mothers have revealed a paradoxical experience of maternity leave: while extended leave enhances maternal and child well‐being, it also raises concerns about job insecurity and career progression. The inconsistent implementation of family‐friendly policies such as flexible working hours, accessible childcare services, and job protection further complicates reproductive decision‐making [20, 21, 22].

International qualitative studies emphasize that affective and experiential factors such as positive reproductive memories, sibling bonding, and religious convictions can significantly shape fertility decisions, sometimes overriding structural constraints [23, 24]. In the Iranian context, although some studies have addressed religious and cultural influences [25, 26], few have examined how these factors intersect with employment realities and institutional support [21, 27]. Despite increasing interest in fertility behavior, regional and global comparative engagement remains limited. Few studies incorporate gender‐sensitive labor policies into fertility analysis, especially in middle‐income countries like Iran [28, 29].

Therefore, the current study explores the motivations for continued childbearing among Iranian working mothers with two biological children, examining how personal values, emotional experiences, and perceived social responsibilities interact with employment conditions. The findings aim to inform culturally sensitive and gender‐responsive population policies.

## 2 Methods

### 2.1 Study Design

This study employed a qualitative design using conventional content analysis as described by Graneheim and Lundman [30]. This approach is suitable for exploring participants' lived experiences and inductively deriving meaning in contexts where theoretical frameworks are limited or exploratory insight is prioritized.

### 2.2 Study Setting

The research was conducted at Kerman University of Medical Sciences in southeastern Iran between September and December 2023. Interviews were held in private locations chosen by participants to ensure comfort and confidentiality.

### 2.3 Sampling and Participants

A purposive sampling strategy with maximum variation was used to recruit working mothers affiliated with Kerman University of Medical Sciences. Inclusion criteria were: (1) being married for at least 5 years, (2) having two or more biological children, (3) being under 49 years of age, and (4) expressing willingness or intent to have an additional child within the next 5 years.

### 2.4 Data Collection

Semi‐structured, in‐depth interviews were conducted by trained researchers. Each session lasted 30–40 min and was audio‐recorded with participant consent. Field notes were taken to capture non‐verbal cues and contextual observations. An interview guide was developed and reviewed by qualitative experts. Key questions included:

“What motivated you to consider having another child?”

“What factors influence your family size decisions?”

“How do social or religious values shape your views on childbearing?”

Follow‐up questions were adapted based on participant responses to ensure depth and relevance.

### 2.5 Data Analysis

Data were analyzed using a hybrid qualitative approach that combined conventional content analysis and inductive thematic analysis to ensure both depth and flexibility in interpretation. The analytic process was guided by the five‐step method proposed by Graneheim and Lundman [30] and complemented by Braun and Clarke's six‐phase framework for thematic development [31].

Interview recordings were transcribed verbatim and imported into MAXQDA 2020 software to facilitate systematic coding and data management. The analysis began with an immersive reading of transcripts to gain a holistic understanding of participants' narratives. Initial open coding was conducted line by line, with codes generated directly from the data without reliance on pre‐existing categories.

Codes were then grouped into meaningful clusters and categorized based on semantic similarity. Through constant comparison and memo writing, sub‐themes were refined and synthesized into overarching themes that captured the core dimensions of fertility motivations. Thematic refinement involved iterative review and discussion among the research team to ensure conceptual clarity and analytic rigor.

Descriptive percentages reported in the Results were calculated as the proportion of participants who endorsed a given code or subtheme (numerator = number of participants with at least one coded segment; denominator = total sample size). Values were rounded to one decimal place for consistency.

To enhance consistency, a second researcher independently reviewed the coding framework and participated in consensus meetings. Saturation was monitored using a saturation matrix and was considered achieved when three consecutive interviews yielded no new codes or themes. A total of 24 interviews established thematic adequacy, and three additional interviews were conducted to confirm stability and reinforce interpretive validity.

### 2.6 Trustworthiness

To enhance the rigor of the study, Lincoln and Guba's criteria for trustworthiness were applied [32]:

- Credibility: achieved through prolonged engagement, member checking, and peer debriefing. Participants were invited to review summaries of their interviews to validate interpretations.
- Transferability: supported by thick descriptions of participant characteristics and context.
- Dependability: ensured through external audit of coding and theme development by two independent reviewers.
- Confirmability: maintained via reflexive journaling and an audit trail documenting analytic decisions.

These procedures were explicitly implemented and documented to provide a clear audit trail and address concerns about methodological transparency.

## 3 Results

A total of 27 working women participated in the study, offering detailed insights into their reproductive motivations. The thematic analysis revealed four dominant motivational axes, each encompassing specific sub‐themes. The most commonly cited theme was Personal and Religious Beliefs (62.9%), indicating that childbearing decisions were often rooted in faith‐based convictions and emotional family values. Following that, Positive Reproductive Memories and Support (55.5%) reflected the role of familial assistance and emotional comfort during previous pregnancies. About 44.4% of mothers expressed Concerns About Single‐Child Outcomes, such as emotional fragility and social isolation. Lastly, 25.9% referenced Demographic Concerns and Pronatalist Influences, including exposure to media campaigns and appreciation for supportive maternity policies. These distributions illustrate that fertility decisions are embedded in a complex matrix of emotional, cultural, religious, and civic considerations (Table 1).

**Table 1:** The categories and sub‐categories extracted after data analysis.

Main theme	Sub‐theme	Participant quotations
1. Personal and religious beliefs	Religious beliefs	“God provides sustenance for every child”; “Abortion is forbidden in our faith”; “Raising children is part of my spiritual duty.”
	Individual beliefs	“Children give meaning to our lives”; “My son is the source of joy in our family”; “Having more children strengthens family bonds.”
2. Positive reproductive memories and support	Support during previous births	“My husband was always beside me during labor”; “Family members helped with childcare and household tasks”; “The emotional support I received made me confident to have another child.”
	Social and emotional capital in large families	“Growing up with many siblings taught me resilience”; “I want my children to experience the warmth of a big family”; “Large families create stronger social ties.”
3. Concerns about single‐child outcomes	Self‐centeredness and fragility	“Only children may become spoiled”; “They expect too much from parents”; “Without siblings, they lack patience and cooperation.”
	Anxiety of loss	“If I lose my only child, I lose everything”; “The thought of losing one child makes me anxious”; “Having more children reduces this vulnerability.”
	Emotional development and social isolation	“Children without siblings feel lonely”; “They miss opportunities to learn empathy”; “Peer interaction at home is essential for social growth.”
4. Demographic concerns and pronatalist influences	Incentive policies	“Government maternity leave encouraged me to plan another pregnancy”; “Financial support helps families manage expenses”; “Workplace flexibility makes childbearing easier.”
	Media influence and national responsibility	“Media campaigns remind us of our duty to the nation”; “I feel responsible to help prevent population aging”; “Public discourse motivates families to have more children.”

### 3.1 Personal and Religious Beliefs (62.9%)

Participants reported that their fertility decisions were influenced by personal and religious beliefs. These convictions shaped their perspectives on childbearing and provided a framework through which they interpreted its meaning and purpose. Two subthemes were identified within this category: religious beliefs and individual values.

#### 3.1.1 Religious Beliefs (40.7%)

Participants emphasized reliance on religious convictions. Believing that God is the provider, that every child is a divine blessing, that abortion is forbidden, and relying on religious verses were central considerations for them. They stated in this regard:

The Qur'an says, ‘Do not worry about their sustenance; it is My responsibility.’ That verse gave me peace, and I trust in God's provision.

(P2)

My fourth child was unplanned. Many people encouraged me to consider abortion, but my faith did not allow it. For me, it was never an option.

(P11)

I believe every child is a divine gift. Rejecting such a gift feels morally wrong, regardless of the circumstances.” (P7)

#### 3.1.2 Individual Beliefs (22.2%)

Participants emphasized individual beliefs that shaped their fertility decisions. Mothers described children as the strength of the family, the capital of the household, a source of authority, the strong arm of the family, and the cane of old age. In addition, some participants believed that both genders, boys and girls, are valuable in different ways for family life. These convictions motivated several participants to pursue subsequent pregnancies. They stated in this regard:

Really, every child makes the family bond stronger. It causes more love in the family. Every time I look at them, I get good energy. It's as if my life before having children was meaningless.

(P1)

Children are really a blessing. They are the fruit of heaven. I come home from work and my tiredness is relieved by them. It gives me pleasure.

(P7)

After two sons, I hoped for a daughter to balance the emotional dynamic of our home. That hope made me pursue another pregnancy.

(P23)

### 3.2 Positive Reproductive Memories and Support (55.5%)

Participants reported that emotionally positive reproductive experiences, together with strong family‐based support systems, influenced their motivation to continue childbearing. Two subthemes were identified within this category: support during previous births and nostalgic attachment to large family life.

#### 3.2.1 Support During Previous Births (37%)

Participants emphasized that the support they received during pregnancy and parenting was pivotal in shaping their willingness to have additional children. The presence of a robust support system fostered a sense of belonging and cooperation, which not only alleviated maternal anxieties but also instilled confidence in managing the challenges of childbearing. Illustrative quotations underscore this dynamic:

In my previous pregnancies, I never felt alone or under pressure, because my husband was completely by my side. That support made me feel I could have another child.

(P17)

After my second baby, I quickly went back to work, and my mother‐in‐law took care of the baby. Her help was vital; without her, I might have stopped at just one child.

(P20)

One of the primary concerns expressed by mothers was the challenge of managing childbearing and parenting responsibilities alone. Participants highlighted that the assurance of receiving help and support from others reduced this fear and increased their willingness to accept the responsibilities of raising children. As one participant explained:

I knew if I became pregnant again, my family, especially my sisters, would support me. That assurance made me feel I did not have to carry all responsibilities alone and reduced my fear.

(P1)

#### 3.2.2 Social and Emotional Capital in Large Families (18.5%)

Participants expressed a preference for large families, drawing upon their own positive childhood experiences. They highlighted the emotional benefits of sibling relationships, including companionship, mutual support across life stages, and the belief that siblings provide long‐term emotional security in the absence of parents. Representative quotations illustrate this sentiment:

I grew up with two brothers and two sisters. After our mother passed away, we supported each other emotionally. That deep sense of solidarity gave me strength, and I hope my children will build a similar bond.

(P11)

Growing up in a large family meant constant companionship. Even now we gather on weekends, and I want my child to experience the same closeness and unity within our own family.

(P8)

We shared everything: rooms, meals, and memories. The noise and laughter were what made our house feel like home. Even now, when our own home is filled with noise, it reminds me of my childhood, and I want my children to experience that same sense of closeness and belonging.

(P14)

### 3.3 Concerns About Single‐Child Outcomes (44.4%)

Participants expressed apprehensions about the possible emotional, psychological, and social consequences of raising an only child. These concerns were mentioned in several interviews, but should be interpreted as individual perspectives rather than universally shared drivers.

#### 3.3.1 Self‐Centeredness and Fragility (18.5%)

Participants expressed concerns that only children might develop unrealistic expectations or limited resilience. A few mothers worried that the absence of siblings could foster self‐centeredness and reduce children's ability to cope with challenges. Illustrative quotations include:

My daughter was the center of our universe. She grew up thinking everything should go her way. I feared she wouldn't cope when life got tougher.

(P2)

My son expects constant praise and instant solutions. If someone says no, it crushes him. That's why I felt he needed a sibling to balance things out.

(P15)

#### 3.3.2 Anxiety of Loss (14.8%)

Participants expressed anxiety about the possibility of losing their only child. They described this fear as emotionally overwhelming, noting that the absence of siblings intensified their sense of vulnerability. Illustrative quotations include:

Some participants described heightened anxiety about the health and safety of their only child, noting that this vulnerability was emotionally taxing:

My colleague lost his only child in an accident. It was very painful. He would not have any more children. I was always worried about losing the only fruit of life. Having only one child causes these worries.

(P6)

I remember once he got a serious illness. I was very scared. What would I do if something happened to him? After all, when you have two or three children, you don't think about these things so much.

(P24)

#### 3.3.3 Emotional Development and Social Isolation (11.1%)

Participants expressed concerns about the emotional development and possible social isolation of their only child. They noted that the absence of siblings might limit opportunities for companionship, sharing, and social learning. Illustrative quotations include:

My child mostly talks to adults. Without siblings, I feel he's missing the emotional training that happens naturally at home.

(P21)

He'll never know what it's like to argue, reconcile, and build shared memories with a sibling.

(P9)

### 3.4 Demographic Concerns and Pronatalist Influences (25.9%)

Participants referred to broader demographic issues and pronatalist influences. In a smaller subset of interviews, participants mentioned these perspectives. Although they were not dominant, they demonstrate how some mothers linked their personal fertility decisions to wider social and policy contexts.

#### 3.4.1 Incentive Policies (18.5%)

Participants mentioned that supportive policies such as extended maternity leave, reduced work hours, or workplace flexibility encouraged them to consider additional pregnancies. Illustrative quotations include:

Because the maternity leave was extended, I did not have to return to work quickly. This period of calm allowed me to consider having another child.

(P18)

After my daughter's birth, I was allowed to transfer to a closer office, which made me feel valued rather than penalized for being a mother.

(P5)

#### 3.4.2 Media Influence and National Responsibility (7.4%)

Participants reported that exposure to media campaigns and public discourse on population decline shaped their sense of civic responsibility. Although these accounts were limited, they reflected how some mothers linked personal fertility decisions to broader national concerns. Illustrative quotations include:

I heard about the ‘population black hole’ on television and became deeply concerned. This apprehension led me to reconsider limiting my family size to only two children.

(P26)

It's not just personal, it's about our future. I felt morally responsible to have more children.

(P10)

## 4 Discussion

Iran, like many countries undergoing rapid socio‐demographic transitions, is experiencing a marked decline in fertility. The total fertility rate has fallen below replacement levels, raising concerns about population aging and long‐term demographic sustainability [33, 34]. While macroeconomic and institutional factors have dominated much of the discourse, this study explores the micro‐level motivations of working mothers who consider having additional children after two births. Their narratives reveal a complex interplay of emotional, cultural, religious, and structural influences that shape reproductive intentions.

Religious and personal beliefs were central to fertility decisions. Many participants described children as “divine blessings,” and motherhood as a spiritually enriching vocation. These sentiments are consistent with global patterns linking religiosity to higher fertility intentions [35, 36]. In Iran, studies by Esmaeili et al. [37] and Khadivzadeh and Irani [38] show that religious narratives often imbue childbearing with moral and spiritual significance, reinforcing communal norms around family formation. Faith‐based convictions helped participants manage anxieties about financial and health concerns [25, 39]. Overall, these findings demonstrate that religiosity and personal convictions act as powerful cultural drivers of fertility intentions, highlighting the interplay between spiritual identity and structural conditions across societies.

Most participants recalled positive reproductive experiences, describing previous pregnancies as emotionally and practically supported by spouses and extended family members. This perceived availability of help reduced psychological and logistical burdens, reinforcing confidence in continued childbearing. These findings align with Vignoli et al. [40], who found that supportive family environments reduce caregiving stress and positively influence fertility outcomes. Iranian studies echo this dynamic, emphasizing the role of familial support in shaping fertility decisions [41, 42]. Grandparents were often described as providing childcare, housing, and financial assistance, creating a multigenerational safety net that facilitated parenting. Older siblings also contributed to caregiving, reducing reliance on passive tools like screen media and enhancing emotional development [43, 44]. Overall, these findings highlight that multigenerational family support serves as a key structural determinant of reproductive confidence, reducing stress and fostering resilience across cultural contexts.

Our findings revealed that many participants expressed nostalgia for large families, recalling the emotional reassurance, solidarity, and cooperative spirit that multiple siblings provided. Mothers emphasized that growing up in larger households fostered resilience and modest expectations, which eased parenting responsibilities and strengthened family cohesion. In contrast, some literature highlights the economic strain of larger families, particularly in lower‐income households where additional children may exacerbate financial hardship and contribute to fertility decline [45, 46]. This apparent contradiction reflects regional variation in socioeconomic status and the subjective framing of “crowding” versus “connectedness” within households [47]. Overall, these findings suggest that nostalgia for large families operates as a culturally contingent motivation, simultaneously evoking ideals of solidarity and concerns of economic strain, underscoring the need for population policies that are sensitive to diverse family experiences.

Participants in our study noted that single children often face heightened expectations of filial piety. Leung and Shek [48] show that filial piety among Chinese only children can foster resilience but also psychological stress when caregiving obligations intensify, while Kyeong et al. [49] argue that filial piety serves both as a cultural anchor and a potential burden. In contrast, Li et al. [50] and Luna et al. [51] report that only children frequently demonstrate adaptability and social competence comparable to peers with siblings. Overall, filial expectations in single‐child households appear culturally contingent, reinforcing duty and stress in some contexts, yet balanced by adaptability in others, highlighting the need for comparative interpretation of fertility motivations.

Mothers, particularly those who were only children themselves, emphasized that sibling absence may heighten emotional vulnerability and anxiety of loss. Sochos and Aleem [52] found that parental attachment style strongly influences bereavement adjustment, with only children often reporting greater dependency and intensified grief. Similarly, Santos et al. [53] showed that childhood anxiety and depression are more pronounced among children lacking sibling support networks. Yet, other evidence suggests that strong parental and community support can mitigate these risks [54, 55]. Taken together, anxiety of loss in single‐child households reflects how family and cultural context shape vulnerability, underscoring the importance of contextualized analysis in fertility research.

Concerns about limited empathy and reduced resilience were linked to restricted peer interaction in single‐child households. Thompson et al. [56] found that social isolation in childhood is associated with poorer mental health outcomes, including depression and conduct problems. However, Luna et al. [51] reported that only children can develop strong adaptability and social skills when supported by family and community structures. Overall, emotional development among only children is highly context‐dependent, requiring cautious interpretation across cultural settings and emphasizing the role of institutional and familial support in shaping outcomes.

Workplace policies such as extended maternity leave, flexible schedules, and approved transfers were mentioned as facilitators, though not primary motivators. Participants emphasized the importance of accessible childcare and early education infrastructure in reducing parenting stress and enabling maternal employment. This nuance is supported by Fluchtmann et al. [57] and Behrman and Gonalons‐Pons [58], who argue that policy alone rarely drives fertility increases unless aligned with cultural expectations and institutional trust. Iranian research similarly emphasizes the importance of perceived feasibility and institutional responsiveness [59, 60]. Overall, these findings reveal that workplace and childcare policies function as enabling conditions rather than primary drivers of fertility, with their effectiveness hinging on cultural alignment and institutional credibility.

A number of participants referred to media campaigns and official discourse surrounding population aging. For some, these messages evoked a sense of civic responsibility, prompting reflection on childbearing as a contribution to national stability. However, as global studies caution, such messaging tends to reinforce existing reproductive intentions rather than generate new ones unless accompanied by comprehensive and sustained policy support [33, 61]. This pattern indicates that while demographic campaigns can evoke civic responsibility, their impact remains conditional on broader institutional support, revealing the limits of messaging as a standalone driver of fertility behavior.

## 5 Conclusion

This study shows that Iranian working mothers' reproductive motivations are shaped by a complex interplay of emotional, cultural, and spiritual factors rather than purely economic or institutional drivers. Positive memories of prior pregnancies, family and spousal support, religious convictions, and concerns about the psychosocial development of only children emerged as central influences. Demographic messaging and workplace policies acted as secondary reinforcements but did not independently trigger fertility intentions. Overall, these findings highlight the need for population policies that are culturally grounded and sensitive to everyday realities, and call for future mixed‐method research to integrate quantitative breadth with qualitative depth for more responsive planning.

## 6 Limitations

The qualitative design, based on a purposive sample from a single institutional setting, restricts generalizability and reflects subjective narratives rather than causal relationships. Moreover, reliance on self‐reported experiences introduces potential recall and desirability biases, particularly in culturally sensitive domains such as reproductive decision‐making. Future studies could benefit from triangulating qualitative insights with longitudinal or comparative data to better understand how emotional, cultural, and structural factors interact over time.

## Author Contributions

N.A., S.P., and J.F. conceptualized and designed the study, supervised all phases of the research, including formative assessment, instrument development, data analysis, and interpretation. They also contributed to drafting and revising the manuscript. N.N. and N.A. were responsible for data collection, preliminary analysis, and preparation of the initial manuscript draft. All authors reviewed and approved the final version of the article.

## Funding

The authors have nothing to report.

## Ethics Statement

This study was approved by the Ethics Committee of Kerman University of Medical Science with ethics No. 402000412 and Ethic approval Code is IR.KMU.REC.1402.274.

## Consent

All participants provided written informed consent. The purpose, voluntary nature, and confidentiality of the study were explained before each interview. Participants were allowed to withdraw at any stage without consequences.

## Conflicts of Interest

The authors declare no conflicts of interest.

## Transparency Statement

The corresponding author, Sirous Pourkhajoei, affirms that this article is an honest, accurate, and transparent account of the study being reported; that no important aspects of the study have been omitted; and that any discrepancies from the study as planned (and, if relevant, registered) have been explained.

## Data Availability

The datasets generated and analyzed during the current study are not publicly available due to ethical considerations and institutional regulations. However, they may be made available upon reasonable request to the corresponding author, subject to approval by the Ethics Committee and completion of appropriate documentation.
